# Causal attribution from retrospective data in Canada's woodland caribou system

**DOI:** 10.1002/eap.70022

**Published:** 2025-05-01

**Authors:** Steven F. Wilson

**Affiliations:** ^1^ EcoLogic Research Nanaimo British Columbia Canada

**Keywords:** boreal forest, causal analysis, habitat disturbance, necessary and sufficient causation, *Rangifer tarandus caribou*, woodland caribou

## Abstract

Forecasting the benefits of management interventions intended to improve ecological conditions requires a causal understanding of the factors that lead to system change. The causal attribution of a factor is defined as the difference between the outcome observed in the presence of the factor and the outcome that would have been observed in the factor's absence, that is, the counterfactual condition. Estimating this contrast is relatively straightforward, where matched or randomized controls are available to approximate the counterfactual condition. However, researchers must reason retrospectively from observational data where matched or randomized controls are not available. In this case, the challenge of establishing causal attribution is in estimating the true counterfactual, that is, the outcome that would have resulted from the absence of the factor, given that it was present. Causal analysis permits the estimation of counterfactuals from observational data, assuming that the model captures all common causes between exposure and outcome, that the exposure is independent of other factors in the model (i.e., exogenous), and that the exposure causes the same directional change for all units (i.e., monotonic). I estimated retrospectively the causal attribution of habitat‐related factors to recruitment rates in Canada's boreal population of woodland caribou (*Rangifer tarandus caribou*). Aggregate habitat disturbance had low causal attribution (17.6%). Attribution was greater (29.5%) when habitat disturbance was disaggregated into different factors associated with different pathways of caribou decline. The causal attribution of all habitat factors considered nevertheless rarely exceeded 50%, suggesting that there are other systematic and/or stochastic factors that can limit the effectiveness of current habitat‐related recovery actions. More effort is required to understand these factors and how they might be managed to improve the probability of successful caribou recovery.

## INTRODUCTION

A central challenge in conservation is the management of complex systems that face multiple interacting factors (Côté et al., 2016). Understanding the relative effects of factors that cause a system's degradation is essential for designing management interventions to improve its condition (Hobbs & Norton, 1996). The *causal attribution* of a factor is defined as the difference between the outcome observed in the presence of the factor and the outcome that would have been observed in the factor's absence (Yamamoto, 2012). This latter outcome is referred to as the *counterfactual* and can be approximated in experimental settings using randomized or matched controls (Coetzee & Gaston, 2021; Ferraro, 2009). Such observations only approximate counterfactual conditions because the true counterfactual can never be observed; a particular unit is either exposed to a factor or not, but not both (Dawid, 2000).

Unfortunately, even approximated counterfactuals are infeasible when inferences about causes must be drawn retrospectively from only observational data. For example, we often want to determine the cause(s) of a species' decline after it has already happened, and evidence might be largely restricted to a time series of population estimates and the changing states of possible contributing factors. Most commonly, correlative model selection methods are applied to predict the population trend using candidate sets of factors, and the set(s) of these covariates explaining the most variance is generally assumed to have caused the observed trend (Arif & MacNeil, 2022; Burnham & Anderson, 2011). But while such models have explanatory power, there is no a priori reason to assume that they accurately represent causal effects (Addicott et al., 2022; Oliver & Roy, 2015).

While causal analysis (Pearl, 2009) is being increasingly applied in ecology (Kimmel et al., 2021; Larsen et al., 2019; Law et al., 2017; Wilson et al., 2021), the application of counterfactual reasoning to the critical issue of causal attribution has not. Here, I apply counterfactual causal analysis to the issue of habitat disturbance and its effect on the demographic performance of woodland caribou (*Rangifer tarandus caribou*) among boreal subpopulations in Canada. I demonstrate how counterfactuals can be applied to estimate the causal attribution of factors, under strong assumptions, when only non‐experimental, retrospective data are available.

## METHODS

### Identifying causal effects from observational data

Pearl (2009) introduced “do calculus” to distinguish between the simple extrapolation of a correlative relationship, where the probability of an outcome, Y, is predicted from exposure X when it takes the value of x:

(1) $$P\left[Y\;|\;\left(X=x\right)\right],$$

from the situation where a treatment forces the exposure *X* to take the value of *x*:

(2) $$P\left[Y\;|\;\mathrm{do}\left(X=x\right)\right].$$

**FIGURE 1:**
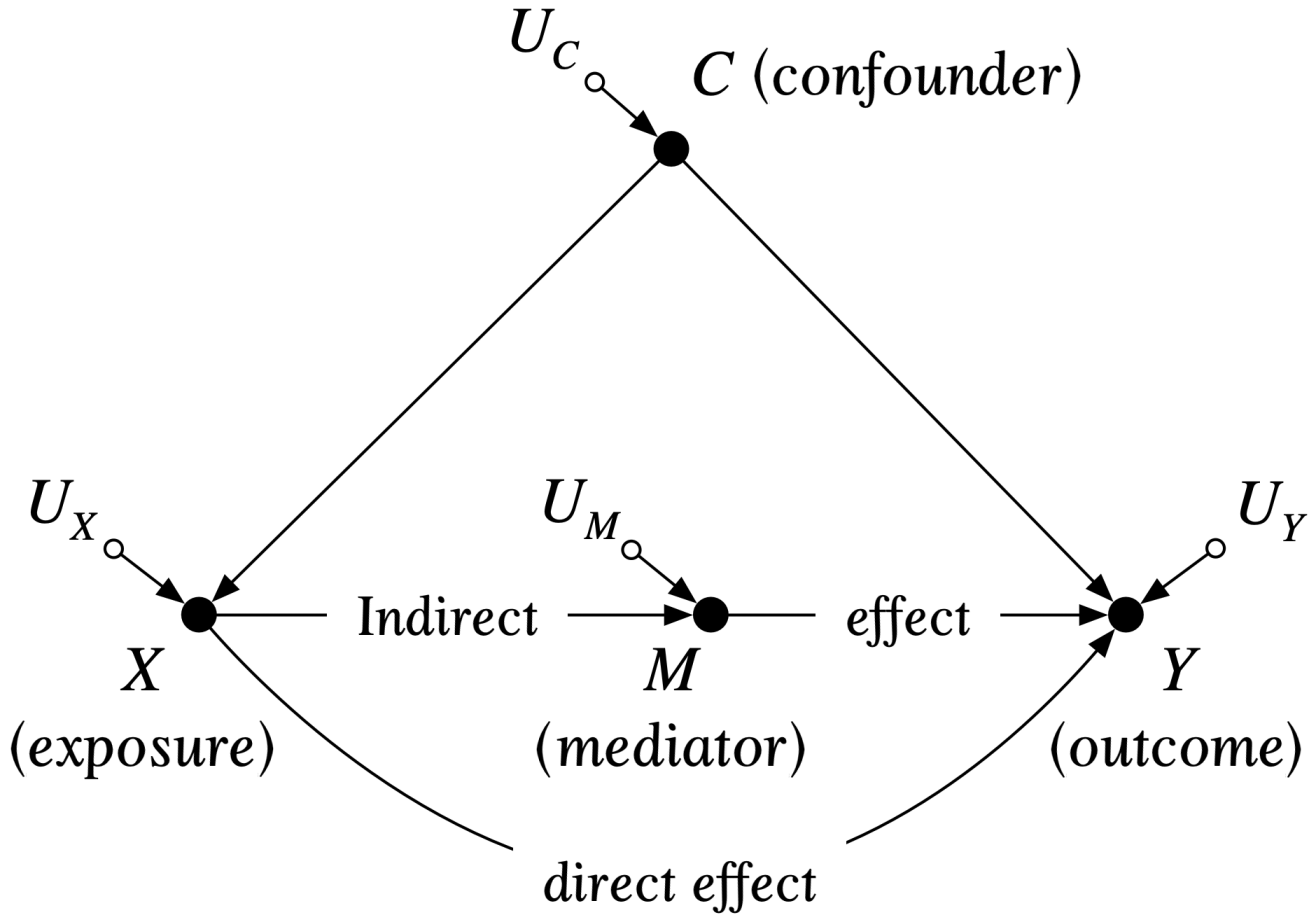
Example of a directed acyclic graph for representing the causal structure of a system, connecting random variables (“nodes”) via arrows (“directed arcs”) to represent causal relationships. An exposure causes an outcome through a direct effect or indirectly via a mediator M. A confounder is a common cause of both the exposure and the outcome. Estimating the causal effect of X on Y in this instance requires statistical adjustment to block the “backdoor” path between exposure and outcome via the confounder C. Uncorrelated U factors represent variation among individuals not explained by the model.

This difference between “observing” (Equation 1) and “doing” (Equation 2) is a key distinction in causal reasoning and distinguishes the first two rungs of Pearl's (2019) “ladder of causation.” While observations of X and Y can be seen to co‐occur, only if intervening to change the value of X changes Y can we claim that the variables are causally related. Moving from “observing” to “doing” also requires more information about the system under investigation. “Observing” a relationship requires only sufficient observations of X and Y to conclude that their correlation coefficient is significantly different from zero. To predict the effect on Y of intervening on X requires either experimental evidence or, in its absence, the application of the tools of causal analysis:

1. A structural causal model (SCM), expressed in Pearl's (2009) framework as a directed acyclic graph (DAG), which specifies all of the causal paths via directed arcs (i.e., arrows) between exposure X and outcome Y, including all observed and unobserved “common causes” between the exposure and outcome (Figure 1), as well as functions defining the relationships between nodes connected by paths.
2. A set of statistical adjustments that block all non‐causal paths without blocking all causal paths, according to a series of causal identification criteria (i.e., conditioning on variables to achieve “d‐separation”) (Grace & Irvine, 2020; Pearl, 2009).

Sometimes, there is no set of adjustments possible to isolate the causal effect of X on Y. For example, a confounder might be known or suspected but unobserved, preventing its adjustment and therefore preventing the blocking of the non‐causal paths. In such cases, the unbiased causal effect on Y of the intervention X = x cannot be estimated from an observational dataset. For example, observing a positive correlation between predator hunting and human‐related conflicts with predators in subsequent years suggests that hunting causes an increase in conflicts (e.g., Teichman et al., 2016); however, without adequate adjustment for the size of the predator population over time, which is rarely known but causally related to both the number of hunter kills and of conflict events, an unbiased causal relationship between hunting and conflicts cannot be inferred.

Pearl (2009) extended causal analysis methods to also reason counterfactually; that is, to make retrospective inferences about how an outcome would have been different if an exposure had been different. The “if” statement presents a hypothetical condition commonly called an *antecedent* (Pearl et al., 2016). A counterfactual analysis estimates how an outcome would have been different had only the antecedent occurred, and all other factors had remained constant. This provides a third level of reasoning and completes Pearl's (2019) “ladder of causation” (Table 1). A counterfactual is conceptually similar to the role of a control group in a randomized controlled trial (RCT). Both aim to provide a basis for understanding what would have happened in the absence of an exposure; however, a control group in an RCT provides a “real world” comparison against the exposed group, while a counterfactual, in Pearl's (2009) logic, is the hypothetical consequence of not being exposed, as inferred from a causal model.

**TABLE 1:** Pearl's (2019) causal hierarchy, distinguishing the types of causal queries and their representation.

Query	Representation	Question	Application
“Observing”	$P\left(Y\;|\;x\right)$	What was Y when we observed X=x?	Interpolating an outcome from a correlative relationship with an exposure
“Doing” (i.e., intervening)	$P\left[Y\;|\;\mathrm{do}\left(x\right)\right]$	What would Y be if we were to set X=x?	Imposing a treatment and observing an outcome
Counterfactual reasoning	$P\left(Y_{x}\;|\;x^{\prime },y^{\prime }\right)$	What would Y have been had X=x rather than the actual treatment of $X=x^{\prime }$, which resulted in the observed outcome $Y=y^{\prime }$	Estimating what an outcome would have been had a different treatment (or no treatment) been applied

### Necessary and sufficient causation

Following Pearl (2022) and using the notation of Hannart et al. (2016), consider the case of a binary exposure to a factor X and a resulting binary outcome Y. The “factual” or real‐world outcome is defined as:

(3) $$p_{1}=P\left(Y=1\;|\;X=1\right),$$

which is the probability that the outcome Y occurred when exposed to the factor X.

In contrast, the counterfactual or “other‐world” outcome is defined as:

(4) $$p_{0}=P\left(Y=1\;|\;X=0\right),$$

which is the probability that Y would have occurred in the absence of X.

Among the units considered in a study, there is a fraction of individuals exposed to the factor (X=1), and the outcome was the result (Y=1). Their counterfactual is the probability that the outcome would not have occurred had exposure to the factor not occurred (expressed here as $Y_{0}=0$). This is known as the probability of necessary causation (PN):

(5) $$\mathrm{PN}=P\left(Y_{0}=0\;|\;Y=1,X=1\right).$$

“Necessary” here means that, although other factors might also have been required for outcome Y to occur, Y would not have occurred *but for* the presence of factor X.

There will also be the fraction of units that were not exposed to the factor (X=0), and the outcome did not occur (Y=0). The counterfactual for this group is the probability that the outcome would have occurred *had* the group been exposed (i.e., $Y_{1}=1$). This is known as the probability of sufficient causation (PS):

(6) $$\mathrm{PS}=P\left(Y_{1}=1\;|\;Y=0,X=0\right).$$

“Sufficient” here means that exposure to X can cause the outcome Y without additional factors but that other factors can also cause the outcome.

From these, the probability of necessary and sufficient causation is then defined as:

(7) $$\mathrm{PNS}=P\left(Y_{0}=0,Y_{1}=1\right).$$

That is, the probability that the outcome Y would have occurred with exposure X and would not have occurred in its absence.

For example, consider wolf reduction programs implemented to increase growth rates of southern mountain caribou in ranges in western Canada (Lamb et al., 2024). In this case, among the fraction of ranges that received the treatment, the probability of necessary causation (PN) expresses the probability that caribou growth rates would not have increased had the wolf reductions not occurred. Among the fraction of ranges not receiving the treatment, the probability of sufficient causation (PS) expresses the probability that growth rates of caribou would have increased had wolf reductions occurred. Combining these counterfactuals into the probability of necessary and sufficient causation expresses the probability that an increase in caribou growth rates would have occurred with exposure to the wolf reduction treatments and would not have occurred in the absence of the treatments.

Tian and Pearl (2000) showed that to reason counterfactually from these quantities from only observational data requires additional assumptions to generate point estimates of causation. First, the exposure X must be “exogenous;” that is, it must have no incoming arcs in the corresponding SCM. Second, the effect of X must be “monotonic,” meaning that the outcome of the exposure for all units must be in the same direction (Manski, 1997). So typically, a factor must be assumed to always have a negative effect among all the exposed, as opposed to having a protective effect for some. If these assumptions hold, then PN, PS, and PNS can be calculated from $p_{1}$ and $p_{0}$ (Equations 3 and 4) as follows:

(8) $$\mathrm{PN}=\max\left\{1-\frac{p_{0}}{p_{1}},0\right\}.$$

(9) $$\mathrm{PS}=\max\left\{1-\frac{1-p_{1}}{1-p_{0}},0\right\}.$$

(10) $$\mathrm{PNS}=\max\left\{p_{1}-p_{0},0\right\}.$$

### Causal attribution

Based only on observational data, we now have the minimum steps and assumptions required to estimate the attribution of an outcome to a specific cause. In summary, these steps are (1) develop and defend an SCM of a system which, at a minimum, includes all observed and unobserved “common causes” between an exposure and outcome; (2) block all non‐casual paths between exposure and outcome via statistical adjustment; and (3) infer the causal effect of exposure X on outcome Y, if the exposure is both exogenous and monotonic.

### Boreal caribou in Canada

The boreal population of woodland caribou (hereafter “boreal caribou” or “caribou”) is distributed throughout much of Canada's boreal forest and is listed under Canada's *Species at Risk Act* as *Threatened* (Environment and Climate Change Canada, 2020). Caribou are essential to the culture and identity of Canada's First Nations people, and their hunting rights are protected under treaties and by Canada's constitution.

Boreal caribou are primarily forest‐dwelling, range in small groups, and do not exhibit the long‐distance seasonal migrations of barren‐ground caribou (*Rangifer tarandus groenlandicus*). They generally occupy low‐productivity spruce (*Picea* spp.) or pine (*Pinus* spp.) forests, which provide partial refugia from predators (primarily wolves [*Canis lupus*]; Environment Canada, 2011). “Disturbance‐mediated apparent competition” is generally considered the most significant threat to caribou persistence (Neufeld et al., 2021), where habitat alteration increases early seral vegetation preferred by other ungulates such as moose (*Alces alces*) and deer (*Odocoileus* spp.), and in turn, predator populations increase and disproportionately affect caribou because of the relatively low productivity of the latter (e.g., Mumma et al., 2018; Serrouya et al., 2015; Superbie et al., 2022; Wittmer et al., 2013). This effect is likely more pronounced where landscape productivity is higher because early seral vegetation is expected to respond to disturbance more vigorously (Neufeld et al., 2021; Serrouya et al., 2021).

Boreal caribou in many parts of Canada have been in decline for decades (Environment Canada, 2011), and a recovery strategy was first published in 2012 and then amended in 2020 (Environment Canada, 2012; Environment and Climate Change Canada, 2020). In addition to identifying the biophysical attributes of critical habitat, the recovery strategy also introduced the management of “disturbed” habitat as a critical habitat requirement. Disturbed habitat was defined as the anthropogenic features identifiable on 1:50,000 Landsat imagery, buffered by 500 m, as well as areas burned within the past 40 years (Environment and Climate Change Canada, 2020). For the recovery strategy, an observed relationship between this measure of disturbed habitat and caribou recruitment (i.e., the ratio of subadults to adult females “recruited” into the breeding population) among a sample of boreal caribou ranges was used to establish a critical habitat requirement of <35% disturbed habitat within each caribou range, a metric which is exceeded among most of Canada's ranges. As noted above and by others (Sleep & Loehle, 2010), managing to such observational relationships risks conflating its predictive power with causal attribution.

### Caribou demographic and habitat data

**FIGURE 2:**
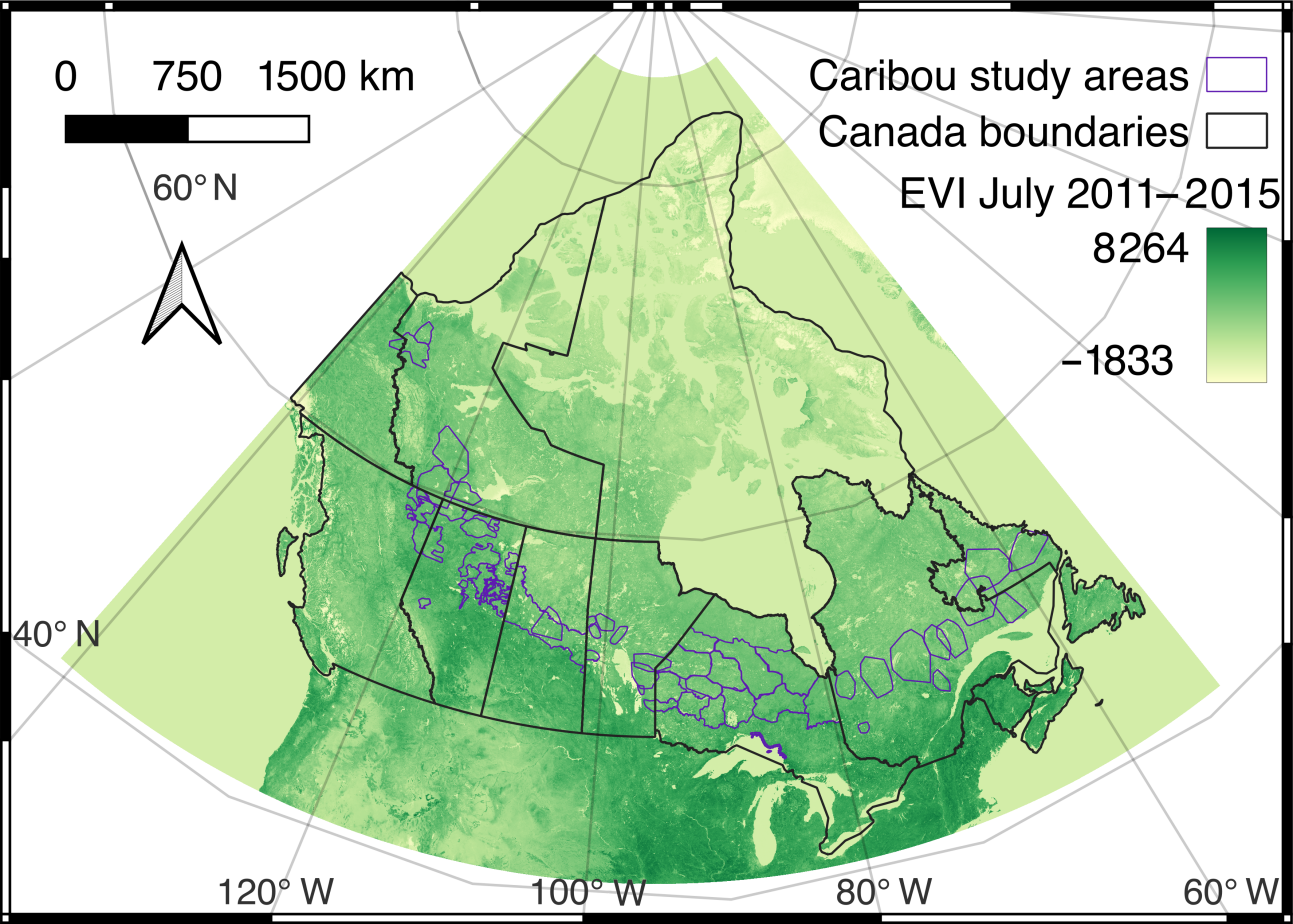
Boreal caribou study areas in Canada and mean Enhanced Vegetation Index (EVI) for July 2011–2015.

For this study, I used caribou demographic, disturbance, and study area boundary data from Johnson et al. (2020), estimating recruitment and study area boundaries from figures where they were not otherwise available. These data covered 58 study areas (Figure 2) throughout Canada's boreal caribou range, with data collected between 1997 and 2017. The analyses were weighted by the number of years of recruitment observations for each study area (159 total observations). Only average recruitment was available for study areas with >1 year observation (Johnson et al., 2020).

I used habitat disturbance data current to 2015 that were assembled by Environment and Climate Change Canada (accessed at: https://open.canada.ca/data/dataset/a71ab99c-6756-4e56-9d2e-2a63246a5e94) using methods described by Pasher et al. (2013). I used the aggregated disturbance metric (i.e., anthropogenic features buffered by 500 m, as well as burned areas <40 years old), but also calculated separately the density of linear features (e.g., roads, seismic lines) in each of the study areas, as well as the percentage of each range covered by forestry cutblocks. As per Pasher et al. (2013), cutblocks were included in the analysis if visible on 1:50,000 Landsat imagery. Because disturbances often overlap, I assigned disturbance type in the following priority: linear features, otherwise polygonal anthropogenic features, otherwise recent fires.

I characterized landscape productivity using MODIS 500‐m Enhanced Vegetation Index (EVI) accessed via Google Earth Engine (https://earthengine.google.com) and Google Colab (https://colab.research.google.com), using a Python script to extract image collections for Canada for July of each year from 2011 to 2015 at a 1‐km resolution (Figure 2). These raster images were then averaged using QGIS 3.2 (QGIS.org, 2023) to characterize landscape productivity. Five years were averaged to address annual variation and cloud cover.

### Assessing causal attribution of habitat disturbance in the caribou system

I posed the following counterfactual query: whether observed declines in the abundance of caribou (as estimated by juvenile recruitment) among subpopulations in study areas with high habitat disturbance would have occurred had the study areas instead had low habitat disturbance and, correspondingly, whether stable‐increasing subpopulations observed in study areas with low habitat disturbance would have remained so had they been exposed to high habitat disturbance.

I used the procedure described in *Boreal caribou in Canada* to estimate: (1) the causal attribution of the aggregate measure of habitat disturbance, under the assumption of a direct and causal effect on caribou recruitment, as presented in Environment Canada (2011) but using the updated data compiled by Johnson et al. (2020) and (2) an alternative model that disaggregated habitat disturbance and landscape productivity to assess different functional pathways affecting the caribou system.

### Aggregate disturbance model

Following Environment Canada (2011, 2012), I used the recruitment rate of 29 calves:100 cows as the binary threshold to classify subpopulations as either stable‐increasing (Y=0) or declining (Y=1; Environment Canada, 2008), and the 35% policy threshold to classify habitat disturbance as either low (X=0) or high (X=1). I also conducted a sensitivity analysis to determine the effect of different aggregate habitat disturbance policies by varying the disturbance threshold ±30% in increments of 5%.

**FIGURE 3:**
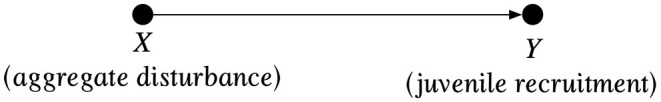
Directed acyclic graph of the aggregate disturbance model, where the relationship between the exposure (*X*) and outcome (*Y*) is assumed to be direct and unconfounded. This is the presumed model informing current policy regarding boreal caribou recovery in Canada (Environment Canada, 2012).

The implied causal model of the aggregate disturbance–recruitment relationship is represented by two nodes (the exposure *disturbance* and outcome *recruitment*) and one directed arc (Figure 3). The observational evidence relating recruitment to habitat disturbance in the context of this assumed model can be considered causal because the exposure is exogenous, and I assume monotonicity (i.e., high disturbance does not benefit caribou recruitment in any study area).

**FIGURE 4:**
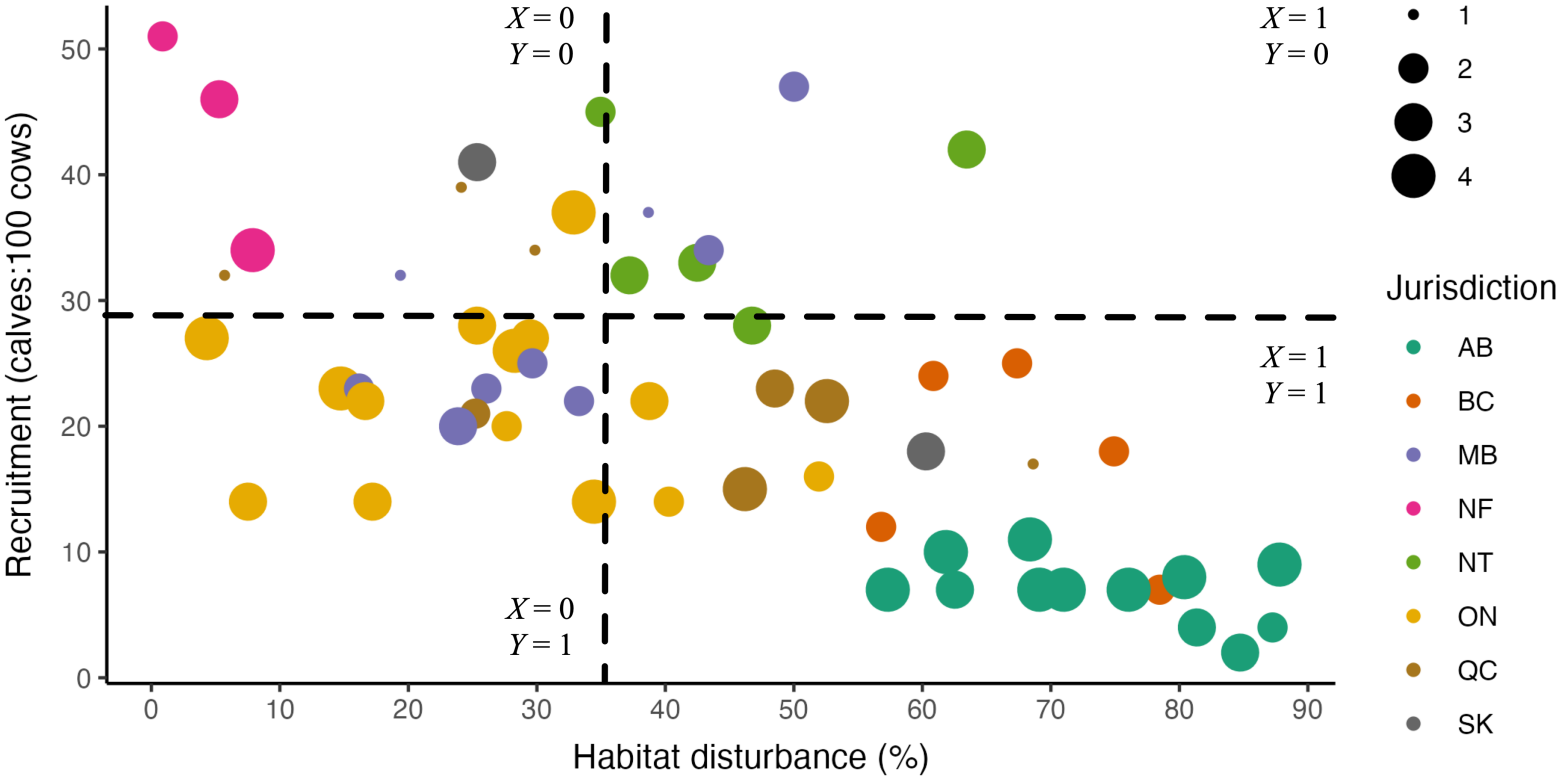
Observational evidence for the relationship between boreal caribou recruitment and aggregate habitat disturbance among study areas, by Canadian jurisdiction, weighted by the number of observations available (years) for each study area. Quadrants distinguish low habitat disturbance (X=0) from high (X=1), and low recruitment (Y=1) from high (Y=0).

I parameterized the SCM as a deterministic Bayesian network model, scripted using the R (version 4.4.2; R Core Team, 2024) package bnlearn (version 5.0.1; Scutari and Ness, 2020) using the binary‐coded data (Figure 4). A Bayesian network codes conditional dependencies among discretized variables, as represented by nodes connected by directed arcs in a DAG (Pearl, 1988). In this instance the binary outcome (high versus low recruitment) was entirely determined by relative frequencies of high or low habitat disturbance. I set evidence in the network model on high disturbance (X=1) and observed the probability associated with low recruitment (Y=1) to calculate the factual probability $p_{1}$ (Equation 3). Here, $p_{1}$ is equivalent to the proportion of observations in the lower right quadrant of Figure 4 in relation to the total number of observations in the lower and upper right‐hand quadrants.

To calculate the counterfactual probability $p_{0}$ (Equation 4), I set evidence in the network model on low disturbance (X=0) and again observed the probability associated with low recruitment (Y=1). Here, $p_{0}$ is equivalent to the proportion of observations in the lower left quadrant of Figure 4 in relation to the total number of observations in the lower and upper left‐hand quadrants.

With these factual and counterfactual probabilities, I then used Equations ((8), (9), (10)) to calculate the probabilities of necessary, sufficient, and necessary and sufficient causation.

### Disaggregated disturbance and landscape productivity model

I developed an alternative causal hypothesis to explain caribou recruitment rates based on separate sources of habitat disturbance (i.e., linear features, cutblocks, and fire) and landscape productivity.

I selected thresholds to distinguish high from low disturbance for the disaggregated model to best distinguish the univariate relationships. There were no observations of high recruitment (i.e., ≥29 calves:100 cows) in study areas with a density of >0.2 km/km^2^ linear features, so I used that threshold to distinguish high linear feature density from low. The same was true where >10% of a study area was classified as forestry cutblocks. For recent fires, all but one study area with high recruitment was associated with a condition in which <40% of the area had burned within 40 years (Dehcho North, NT). For primary productivity, only one study area with high recruitment was associated with an average EVI > 3500 (Bloodvein, MB). These values maximized $p_{1}$ for the respective factors and therefore the causal attribution.

**FIGURE 5:**
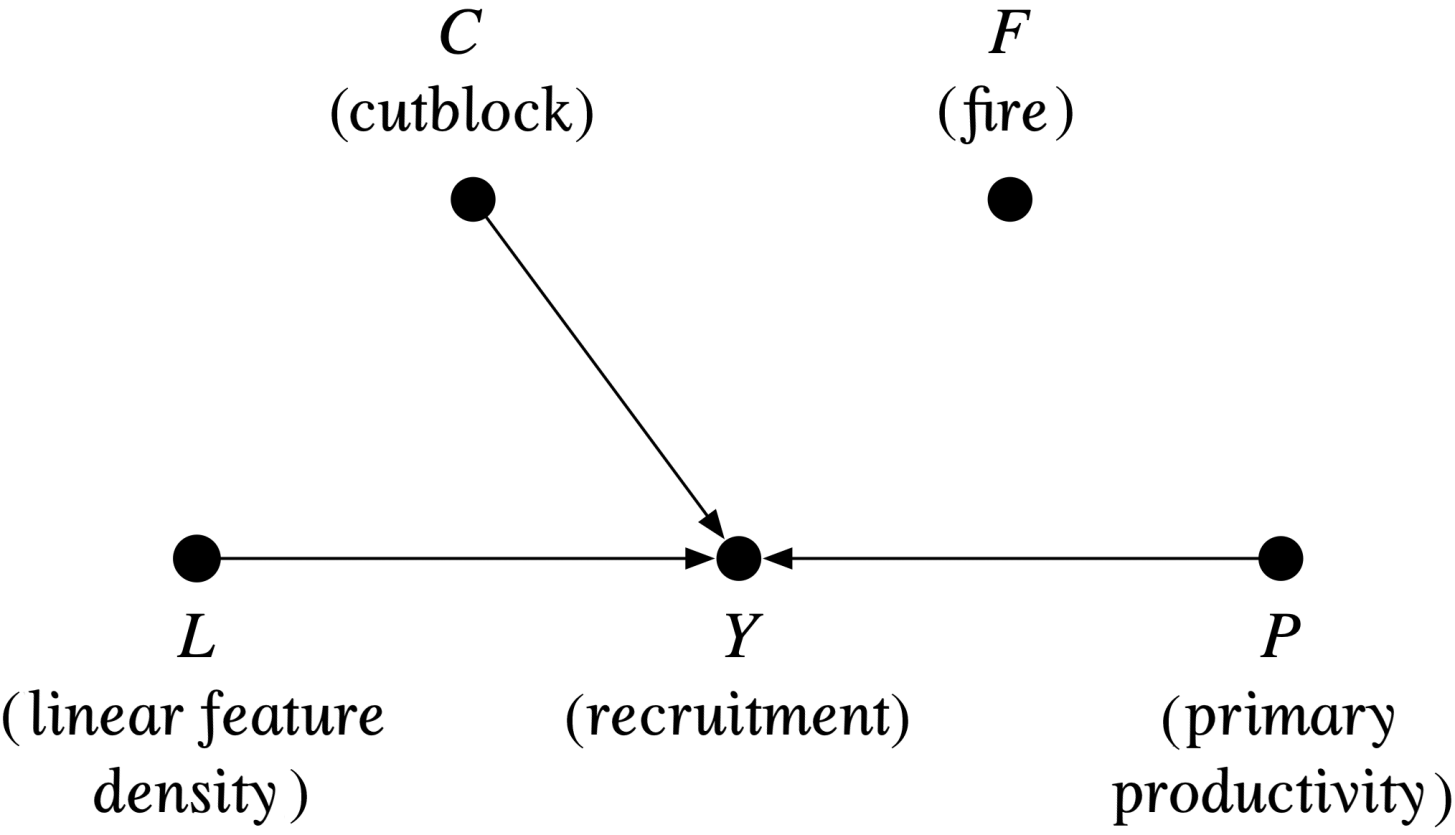
Structure of the disaggregated disturbance and landscape productivity model. Fire was excluded due to low conditional mutual information with the recruitment outcome node.

I hypothesized a simple causal structure with all four factors as independent parents of recruitment and fitted data to the network. Inclusion of fire as a factor generated empty cells in the conditional probability table (regardless of the threshold), and this factor had the lowest conditional mutual information with the target recruitment node, so I removed the causal arc (Figure 5). This causal network met the criterion of exogeneity (i.e., no incoming arcs to the exposures) and I assumed monotonicity in the effect of the factors. I then used Equations (3, 4, (8), (9), (10)) to calculate the probabilities of necessary, sufficient, and necessary and sufficient causation.

## RESULTS

### Aggregate disturbance model

Based on the aggregate disturbance model, the probability that high habitat disturbance (>35%) was necessary (PN) to cause low recruitment was 20.8% (Table 2), lower than the probability that it was sufficient (PS = 53.7%). Collectively, the probability that high habitat disturbance was both necessary and sufficient (PNS) to cause low recruitment was 17.6%.

**TABLE 2:** Summary of causation probabilities for the aggregated and disaggregated disturbance models.

Model	Conditioning	Evidence	Factual probability of event (*p*_1_)	Counterfactual probability of event (*p*_0_)	Probability of necessity (PN %)	Probability of sufficiency (PS %)	Probability of necessity and sufficiency (PNS %)
Aggregate disturbance	None	Treatment <65% undisturbed	0.848	0.672	20.8	53.7	17.6
Primary productivity	None	>3500	0.980	0.677	31.0	93.9	30.3
Separate disturbance	None	Linear >0.02	1.000	0.742	25.8	100.0	25.8
		Cutblocks >10%	1.000	0.803	19.7	100.0	19.7
		Cutblocks >10%, Linear >0.02	1.000	0.705	29.5	100.0	29.5
	High EVI	Linear >0.02	1.000	0.970	3.0	100.0	3.0
		Cutblocks >10%	1.000	0.977	2.3	100.0	2.3
		Cutblocks >10%, Linear >0.02	1.000	0.966	3.4	100.0	3.4
	Low EVI	Linear >0.02	1.000	0.515	48.5	100.0	48.5
		Cutblocks >10%	1.000	0.631	36.9	100.0	36.9
		Cutblocks >10%, Linear >0.02	1.000	0.446	55.4	100.0	55.4

Abbreviation: EVI, Enhanced Vegetation Index.

**FIGURE 6:**
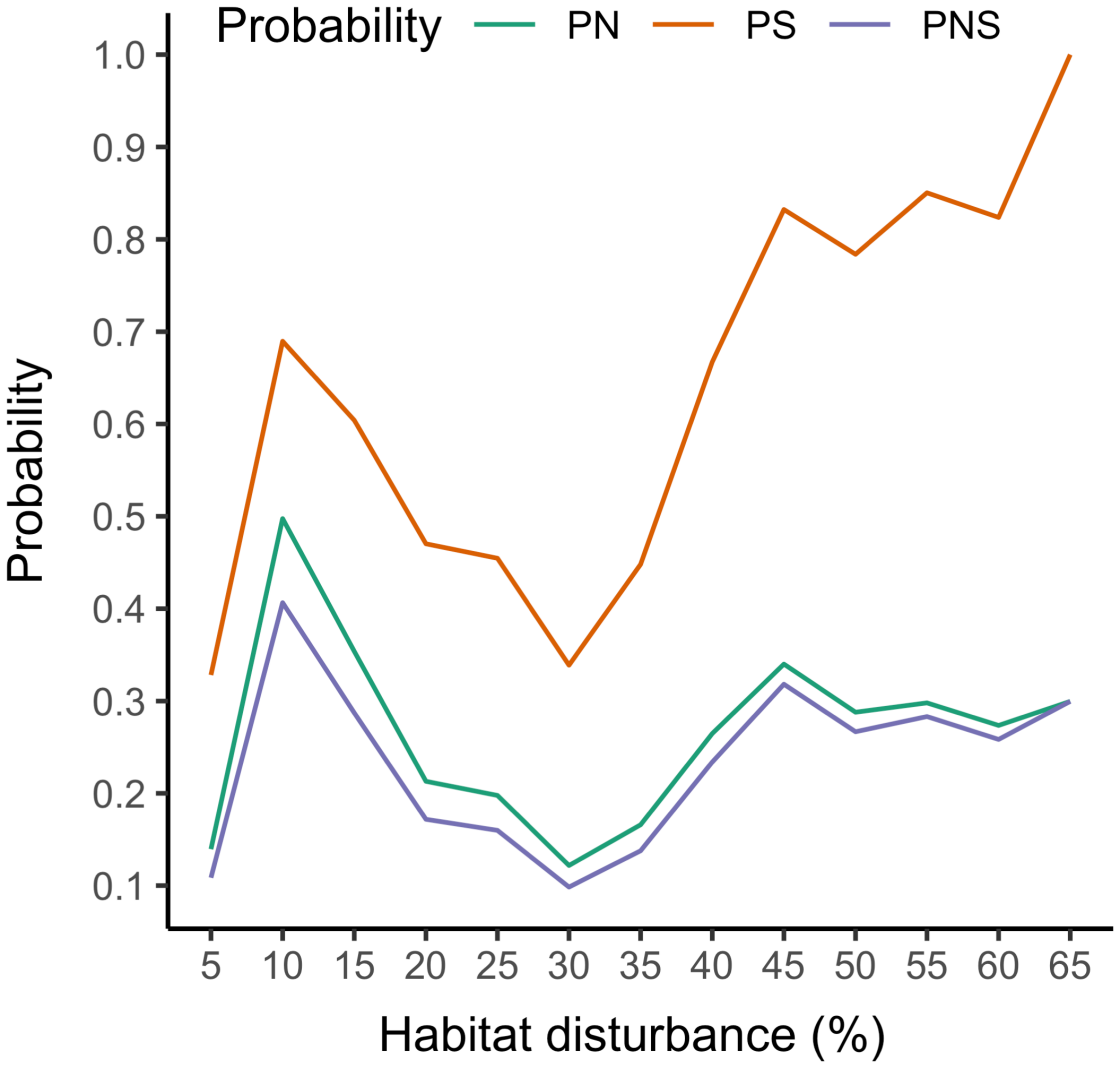
Results of the sensitivity analysis for the aggregate disturbance model, illustrating how the probabilities of causation (PN, probability of necessary causation; PS, probability of sufficient causation; PNS, probability of necessary and sufficient causation) change with different thresholds of aggregate disturbance.

The probabilities of causation were sensitive to the habitat disturbance threshold, with minima occurring at 30% (Figure 6). At increasing disturbance thresholds, PS increased to 1 as the number of observations of high disturbance ranges with high recruitment went to 0 above a threshold of 60%. Results at thresholds of 5%–15% were more variable, with small sample sizes and shifting ratios of ranges with high recruitment versus low.

### Disaggregated disturbance and productivity model

High linear feature density (i.e., >0.2 km/km^2^) had a PN of 25.8% (Table 1) and a PS = 100% (given that there were no observations of stable‐increasing subpopulations in ranges with high linear feature density). The PNS was the same as the PN (25.8%) because PS = 100%. The PN and PNS of cutblocks >10% of a range were 19.7%. The cumulative effect of both disturbance sources had a PN and PNS of 29.5%.

Primary productivity, as measured by average July EVI, had a PNS of 30.3%, exceeding that of either high linear feature density or cutblocks >10% of a range. Stratifying by high versus low productivity changed the probabilities of causation. The cumulative effect of linear features and cutblocks (high habitat disturbance) was associated with a low PNS (<3.5%) in high productivity areas. Yet in low productivity areas, high habitat disturbance was associated with a high PNS (55.4%).

## DISCUSSION

Environmental policy analysis requires knowledge about cause‐and‐effect relationships, but experimental evidence is often difficult to acquire (Cucurachi and Suh, 2017). In these circumstances, it is common to conduct correlative analyses of observational data and then speculate about causal relationships. But basing management interventions on causal speculation can be costly, particularly if an intervention has a high direct or opportunity cost and the desired outcome is not realized because of underlying flaws in the causal logic. The central question in reasoning retrospectively about causality in ecological systems is whether an observed adverse outcome would have been different if an exposure had not occurred (Coetzee and Gaston, 2021). Such estimates provide a stronger basis for designing policy interventions to reverse an adverse outcome. As demonstrated here, advances in causal analysis provide analytical tools to estimate retrospectively the causal contribution of different factors in the absence of experimental data.

Of course, no analysis of observational data can match the power of a randomized control trial for establishing causality (Cartwright, 2010), and a causal model based on only observations remains, at its core, an analysis of correlations, to which well‐known cautions in interpretation apply (Cliff, 1983). Moving from predictive to causal models, in part, requires a shift from these cautions to explicit assumptions (e.g., the caution of confounding in the interpretation of correlational models becomes the assumption of exogeneity in causal models). As a result, the acceptance of inferences derived from causal models relies as much on the transparency and reasonableness of their assumptions as on their statistical diagnostics. I suggest that considering models as causal hypotheses and evaluating their consequences under strict assumptions is an improvement over implicitly assuming causality in statistical relationships. Further, I suggest that PN, PS, and PNS are simple and intuitive estimates of the counterfactual implications of such hypotheses and allow researchers to explore retrospective questions of causal attribution with available observational evidence.

In this study, the counterfactual query posed was whether observed declines in the abundance of caribou among subpopulations in study areas with high habitat disturbance would have occurred had the study areas instead had low habitat disturbance and, correspondingly, whether stable‐increasing subpopulations observed in study areas with low habitat disturbance would have remained so had they been exposed to high habitat disturbance. While others have extrapolated regression‐based models to infer retrospectively or prospectively the population responses of caribou under alternative management regimes (e.g., Johnson et al., 2020; Stewart et al., 2020; Serrouya et al., 2011), none have adopted an explicit causal‐counterfactual approach as I have here.

With the implied causal model and current habitat disturbance policy threshold of 35%, aggregate habitat disturbance was found to have low causal attribution (PNS = 17.6%), suggesting that the current recovery objective to reduce aggregate disturbance to <35% has on average a low probability of successfully increasing recruitment to a level consistent with stable‐increasing populations. The reason for this low PNS is that, while many subpopulations in high habitat disturbance study areas are indeed in decline, most subpopulations in low‐disturbance conditions are also in decline. As well, there are also stable‐increasing subpopulations in study areas with high habitat disturbance. Observations of these circumstances are contrary to the predicted habitat disturbance–recruitment relationship, and their relative frequencies inform the causal interpretation of the model. In this case, the model had a relatively low PN but higher PS. This suggests that habitat disturbance is far from essential (PN = 20.8%) but can be adequate (PS = 53.7%) to cause low caribou recruitment on its own. It also suggests that there are multiple causal pathways, of which aggregate disturbance is only one.

The probabilities of causation are calculated from binary inputs and therefore are subject to threshold effects. In this study, percentages of habitat disturbance both below and above the 35% policy threshold were associated with higher probabilities of causation. In practice, the probabilities of causation are minimal when $p_{1}$ and $p_{0}$ are most similar because evidence for a causal relationship is necessarily weak when an outcome occurs counterfactually at a similar frequency as factually. As the probability of a counterfactual outcome declines relative to the factual outcome, the probabilities of causation increase. The methods to extend the probabilities of causation to continuous variables are emerging (Kawakami et al., 2024).

Revising the causal model to disaggregate sources of habitat disturbance into different pathways to decline suggested that there are disturbance conditions that, based on currently available data, are incompatible with stable‐increasing caribou populations; specifically, linear feature densities of >0.2 km/km^2^ and/or areas where forestry cutblocks comprise >10% of a range's area. Both thresholds were associated with a PS = 100%, indicating no evidence that caribou subpopulations can be stable in conditions exceeding these thresholds.

Linear features and forestry cutblocks are generally associated with different pathways to decline in the caribou system, with cutblocks generating early seral vegetation assumed to drive apparent competition and linear features providing travel corridors that increase the permeability of habitats that otherwise serve as predator refugia for caribou (e.g., DeMars & Boutin, 2018; Dickie et al., 2017; Mumma et al., 2018). These two factors have been characterized broadly as the “numerical” and “functional” responses of the predator–prey system to human‐caused habitat alteration, and this analysis suggests that linear feature density has the stronger causal attribution (PNS of 25.8% vs. 19.7%), with the cumulative effect of exceeding both thresholds being higher (PNS = 29.7%). This is the first study to assign independent causal attribution to these factors but is consistent with the conclusions of Mumma et al. (2018), who found, in northeast British Columbia, more support for the direct effect of linear features on caribou−wolf overlap than for numeric or spatial apparent competition.

The proportion of study areas recently burned by wildfire was not a significant factor in the analysis. Recent studies suggest that fire may not be as important a driver in boreal caribou population declines as originally thought (DeMars et al., 2019; Johnson et al., 2020; Neufeld et al., 2021). However, this might not hold if climate change increases the frequency of extreme fire behavior in Canada as predicted (Wang et al., 2015).

Similar to Serrouya et al. (2021) and Neufeld et al. (2021), I found that primary productivity was a significant factor in the caribou system. The probability of necessary and sufficient causation of primary productivity (30.3%) was similar to that for the combined anthropogenic sources of linear features and cutblocks (29.5%). Causal attribution was higher for anthropogenic disturbance sources in areas of low primary productivity, suggesting that factors other than habitat disturbance may be important in more productive habitats. This could include white‐tailed deer (*Odocoileus virginianus*) expansion independent of significant habitat change (e.g., through climate warming; Dawe & Boutin, 2016), which also contributes to apparent competition.

Habitat disturbance is often referred to as the “ultimate” cause of caribou declines because of the cascading effects on vegetation and predator–prey changes it precipitates (e.g., Baillie‐David et al., 2024; DeMars et al., 2023; Festa‐Bianchet et al., 2011; Nagy‐Reis et al., 2021), leading some to conclude that habitat recovery will be sufficient to obviate the need for intensive, population‐based actions such as predator control (Environment and Climate Change Canada, 2020; Lamb et al., 2024). However, the causal attribution (PNS) of the disturbance‐related factors I considered was only 29.5% range‐wide, suggesting that such a conclusion may be premature and that there are unobserved systematic (implying a different causal model) or stochastic factors operating in the boreal caribou system that might limit the effectiveness of habitat restoration or natural regeneration in re‐establishing self‐sustaining caribou populations. These factors could include spatial or temporal variation or shifts in forage supply, diseases/parasites, hunter harvest, displacement, or others (DeMars et al., 2023). Successful caribou recovery will require a better understanding and management of these other factors causing caribou to decline.

## CONFLICT OF INTEREST STATEMENT

The author declares no conflict of interest.

## Data Availability

Data are available in appendix S1: supplementary analysis of Wilson et al. (2021) at https://doi.org/10.1016/j.biocon.2021.109370. Novel code and datasets (Wilson, 2025) are available in the Open Science Framework (OSF) repository at http://doi.org/10.17605/OSF.IO/JDZGA.
